# Healthcare disparities in cardio oncology: patients receive same level of surveillance regardless of race at a safety net hospital

**DOI:** 10.1186/s40959-020-00080-w

**Published:** 2021-01-25

**Authors:** Crystal B. Chen, Raj K. Dalsania, Eman A. Hamad

**Affiliations:** 1 Department of Medicine, Temple University Hospital, Philadelphia, PA USA; 2 Department of Cardiology, Temple Heart and Vascular Institute, Section of Advanced Heart Failure and Transplantation, Temple University Hospital, 3401 N Broad Street, Parkinson Pavilion, 9th Floor, Philadelphia, PA 19140 USA

**Keywords:** Cardio-oncology, Heart failure, Chemotherapy, Cardiotoxicity, Disparity, Income, ZIP code, Referral

## Abstract

**Background:**

Cardiotoxicity remains a dreaded complication for patients undergoing chemotherapy with human epidermal growth factor (HER)-2 receptor antagonists and anthracyclines. Though many studies have looked at racial disparities in heart failure patients, minimal data is present for the cardio-oncology population.

**Methods:**

We queried the echocardiogram database at a safety net hospital, defined by a high proportion of patients with Medicaid or no insurance, for patients who received HER2 receptor antagonists and/or anthracyclines from January 2016 to December 2018. Patient demographics, clinical characteristics, and treatment outcomes were collected. Based on US census data in 2019, home ZIP codes were used to group patients into quartiles based on median annual household income. The primary end point studied was referral rate to cardiology for patients undergoing chemotherapy.

**Results:**

We identified 149 patients who had echocardiograms and also underwent treatment with HER2 receptor antagonists and/or anthracyclines, of which 70 (47.0%) were referred to the cardio-oncology program at our institution. Basic demographics were similar, but white patients were more likely to live in ZIP codes with higher income quartiles (*p* < 0.00001). Comparing between racial groups, there was no statistical difference in the percentage of patients that had a reduction in ejection fraction (EF) (*p* = 0.75). There was no statistical difference between racial groups in the number of cardiology or oncology appointments attended, number of appointments cancelled, average number of echocardiograms received, additional cardiac imaging received. Black patients were more likely to receive ACEI/ARB post chemotherapy (*p* = 0.047). A logistic regression model was created using race, age, gender, insurance, income quartile by home ZIP code, comorbidities (hypertension, hyperlipidemia, coronary artery disease, arrhythmia, diabetes mellitus, smoking, family history, age > 65), procedures (coronary stents, cardiac surgery), medications pre-chemotherapy, cancer type, cancer stage, and chemotherapy. This model found that there was an increased referral rate among patients from higher income quartiles (*p* = 0.017 for quartile 3, *p* = 0.049 for quartile 4), patients with a history of hypertension (*p* < 0.0001), and patients with breast cancer (*p* = 0.02).

**Conclusions:**

The results of this study suggest that patients of our cardio-oncology population at a safety net hospital receive the same level of surveillance and treatment, and develop drop in ejection fraction at similar rates regardless of their race. However, patients that reside in ZIP codes associated with higher income quartiles, with hypertension, and with breast cancer, are associated with increased rate of referral.

## Introduction

Over the past decade, cancer treatment has advanced dramatically. However, the ability to effectively apply these treatments has been limited by patient tolerability. Chemotherapy induced cardiotoxicity is the second leading cause of morbidity and mortality among cancer patients, a close second after secondary malignancies [1]. The most common cardiotoxic agents are HER2 antagonists (trastuzumab being the most common) and anthracyclines, with an incidence rate of 7–28% for HER2 antagonists and 8–26% for anthracyclines [2]. There are many definitions of cardiotoxicity; the current working definition posed by the Cardiac Review and Evaluation Committee of Trastuzumab-associated Cardiotoxicity is a symptomatic reduction in left ventricular ejection fraction (EF) by at least 5% to less than 55% or an asymptomatic reduction in EF by at least 10% to less than 55% [3].

Cardiotoxicity in chemotherapy patients has proven to be difficult to manage. Many of the unknowns regarding toxicity include its pathophysiology, determining which patients may be more prone to cardiotoxicity, and the appropriate treatment of cardiotoxicity in a way that is both safe for patients and allows for completion of chemotherapy. Studies looking at risk factors for chemotherapy induced cardiotoxicity have shown varying results. Though some studies find no correlation between known cardiovascular risk factors and the development of cardiotoxicity, [4–6] there have been several other studies with evidence of such correlation, specifically with hypertension (HTN), hyperlipidemia (HLD), diabetes mellitus type II (DM II), women above 65 years of age, and a positive family history of cardiovascular disease or anthracycline or trastuzumab induced cardiotoxicity [7–11].

Monitoring for cardiotoxicity is crucial for patients who are on HER2 antagonists and anthracyclines. Imaging strategy involves using transthoracic echocardiogram (TTE) to get a baseline EF prior to the patient undergoing chemotherapy. Subsequent TTEs are used for monitoring purposes during and after treatment. We now have specific echocardiographic parameters such as tissue Doppler derived strain and strain rate measurements that we can use to diagnose and predict the onset of cardiotoxicity. Additionally, there are instances where more advanced imaging modalities such as cardiac MRI or multiple-gated acquisition (MUGA) scan may be indicated on an individual basis. As recommendations by various national organizations vary at the frequency and timing of repeat imaging, the guidelines for cardiotoxicity screening and surveillance remain a gray area [2, 4].

The African American population has been shown to have disproportionately higher death rates related to breast cancer [12]. The reasoning for this is inconsistent across studies, but many contribute it to suboptimal treatment in this racial group [13, 14]. However, there is a paucity of studies on racial disparities in patients who suffer from chemotherapy induced cardiotoxicity. One study looked at rates of cardiotoxicity among females with breast cancer undergoing treatment with trastuzumab and found higher rates of cardiotoxicity in black women compared to white women, resulting in early termination of chemotherapy in black female patients [15]. A similar trend was found in black patients receiving doxorubicin therapy as well [16]. Therefore, enhanced cardiac surveillance and early referral for this population is crucial, especially since guideline-adherent cardiac monitoring was only identified in about 46% of all patients [17]. In this study, we aim to determine whether black patients receiving cardiotoxic chemotherapy were undertreated or had worse clinical outcomes at our institution.

## Material and methods

This is a retrospective analysis in which we queried the echocardiogram database at a safety net hospital, defined by a high proportion of patients with Medicare, Medicaid, or no insurance, for patients who received trastuzumab and/or doxorubicin from January 2016 to December 2018. Patients were excluded if their electronic medical record contained insufficient data. Patient demographics, including patient age, gender, race, and medical insurance were obtained. Home ZIP codes were collected as a surrogate for socioeconomic status. Using the United States Census data in 2019, ZIP codes were used to group patients into quartiles based on median annual household income, with quartile 1 earning $0 - $18,900, quartile 2 earning $19,000 - $32,800, quartile 3 earning $32,900 - $56,000, and quartile 4 earning $57,000 - $130,300. Clinical characteristics included comorbidities, procedures, medications prior to initiation of chemotherapy, cancer type, cancer stage, chemotherapy (trastuzumab and/or doxorubicin), number of echocardiograms received, and EF. As described previously, chemotherapy induced cardiotoxicity was defined as a symptomatic reduction in EF by at least 5% to less than 55% or an asymptomatic reduction in EF by at least 10% to less than 55% [2]. Referral patterns described included whether the patient was referred to the cardio-oncology program, the number of visits, the number of cancelled visits or no-shows, the number of oncology visits, and cardiac imaging and medications received after initiation of chemotherapy.

The aim of the study was to determine whether racial and economic disparities exist in cardio-oncology patients undergoing chemotherapy with trastuzumab and doxorubicin. The primary end point studied was the referral rate of these patients to cardio-oncology or cardiology clinic. Secondary endpoints included the number of cardio-oncology visits, cancelled visits or no shows, number of oncology visits, and cardiac imaging and medications post chemotherapy initiation .

Statistical analysis was performed using t-tests and one-way ANOVA for continuous variables and chi-square tests for categorical variables. A binary multivariate logistic regression model predicting associations with referral rate was generated using univariate risk factors. All statistical analyses were performed using SPSS software.

## Results

We identified 149 patients who received echocardiograms and also underwent trastuzumab and/or doxorubicin therapy. The cohort was predominantly black (*n* = 68, 45.9%), followed by white (*n* = 41, 27.5%), Hispanic (*n* = 33, 22.1%), and other (*n* = 7, 4.7%). The other category included 5 Asians and 2 listed as “others” in the electronic medical record.

### Socio-demographics

**Fig. 1:**
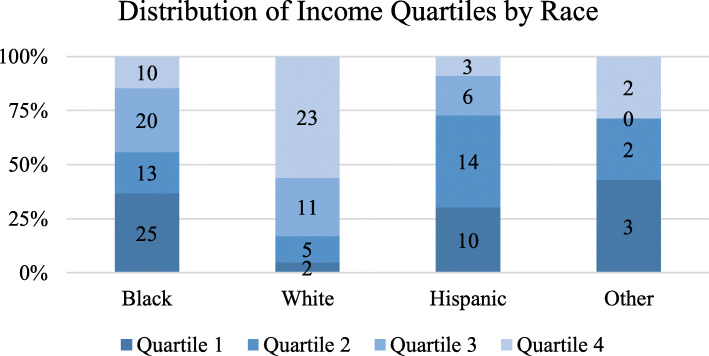
Distribution of Income Quartiles by Race. White patients were more likely to live in ZIP codes with higher median annual household income compared to black, Hispanic, or other races (p < 0.00001)

Baseline socio-demographics were similar between racial groups in terms of age, gender distribution, and medical insurance (Table 1). About half of black patients had private insurance, which is similar to that of whites, Hispanics, and other races (54.4, 56.1, 48.5, 42.9%, respectively, *p* = 0.77). Black, Hispanic, and other race patients were more likely to live in ZIP codes with lower median annual household income compared to white patients (*p* < 0.00001) (Fig. 1). Two (4.9%) white patients live in the lowest quartile of ZIP codes by income, compared to 25 (36.8%) black patients, 10 (30.3%) Hispanic patients, and 3 (42.9%) other race patients (*p* < 0.0001). In contrast, 23 (56.1%) white patients live in the highest quartile of ZIP codes by income, compared to 10 (14.5%) black patients, 3 (9.1%) Hispanic patients, and 2 (28.6%) other race patients.

**Table 1:** Baseline demographics and clinical characteristics of patients by race

	Black (N = 68)	White (N = 41)	Hispanic (N = 33)	Other (N = 7)	*P*-value
**Age at diagnosis (median, [IQR])**	58 (51, 66)	58 (52, 65)	53 (44, 59)	61 (43, 66)	***p*** **= 0.54**
**Gender**					***p*** **= 0.30**
Male	6 (8.8%)	9 (22.0%)	5 (15.2%)	1 (14.3%)	
Female	62 (91.2%)	32 (78.0%)	27 (81.8%)	6 (85.7%)	
**Insurance**					***p = 0.77***
Private insurance	37 (54.4%)	23 (56.1%)	16 (48.5%)	3 (42.9%)	
Medicare	25 (36.8%)	12 (29.2%)	10 (30.3%)	3 (42.9%)	
Medicaid	2 (2.9%)	1 (2.4%)	2 (6.1%)	0 (0.0%)	
No insurance	4 (5.9%)	5 (12.2%)	5 (15.2%)	1 (14.3%)	
**Income by ZIP code**^a^					***p < 0.00001***
Quartile 1	25 (36.8%)	2 (4.9%)	10 (30.3%)	3 (42.9%)	
Quartile 2	13 (19.1%)	5 (12.2%)	14 (42.4%)	2 (28.6%)	
Quartile 3	20 (29.4%)	11 (26.8%)	6 (18.2%)	0 (0.0%)	
Quartile 4	10 (14.7%)	23 (56.1%)	3 (9.1)	2 (28.6%)	
**Comorbidities**					
HTN	46	22	15	3	***p*** **= 0.13**
HLD	18	16	11	2	***p*** **= 0.72**
CAD	4	6	2	0	***p*** **= 0.38**
Arrythmia	5	2	2	0	***p*** **= 0.82**
DM	19	10	9	1	***p*** **= 0.87**
Smoking	33	18	10	1	***p*** **= 0.18**
FH	20	12	7	2	***p*** **= 0.86**
Age > 65	16	11	5	1	**p = 0.60**
**Procedures**					
Coronary stents	1	1	0	0	***p*** **= 0.81**
Cardiac surgery	0	3	1	0	***p = 0.13***
**Medications pre-chemo**					
Beta blocker	11	9	4	1	***p = 0.72***
ACEI/ARB	25	13	7	0	***p = 0.13***
Diuretic	7	1	2	0	***p*** **= 0.41**
Hydralazine	2	0	0	0	***p*** **= 0.49**
Nitrate	0	0	2	0	***p*** **= 0.37**
MRA	0	0	0	0	***p*** **= 1**
CCB	12	3	2	0	***p*** **= 0.26**
Antiarrhythmic	0	0	0	0	***p = 1***
Aspirin	13	8	2	1	***p*** **= 0.35**
Statin	13	10	5	0	***p*** **= 0.76**
**Cancer type**					***p = 0.036***
Breast cancer	57 (83.8%)	24 (58.5%)	24 (72.7%)	5 (71.4%)	
Other cancer^b^	11 (16.2%)	17 (41.5%)	9 (27.3%)	2 (28.6%)	
**Cancer stage**					***p*** **= 0.98**
Stage 1	6 (8.8%)	3 (7.3%)	2 (6.1%)	1 (14.3%)	
Stage 2	32 (47.1%)	18 (43.9%)	18 (54.5%)	3 (42.9%)	
Stage 3	14 (20.6%)	9 (22.0%)	5 (15.2%)	1 (14.3%)	
Stage 4	16 (23.5%)	12 (29.3%)	7 (21.2%)	2 (28.6%)	
**Chemotherapy**					***p*** **= 0.50**
Doxorubicin	24 (35.3%)	22 (53.7%)	15 (45.5%)	3 (42.9%)	
Herceptin	39 (57.4%)	15 (36.6%)	14 (42.4%)	4 (57.1%)	
Doxorubicin and herceptin	5 (7.4%)	4 (9.8%)	4 (12.1%)	0 (0.0%)	
**Ejection fraction**					**p = 0.75**
Decrease^c^	19 (27.9%)	11 (26.8%)	6 (18.2%)	2 (28.6%)	
No decrease	49 (72.1%)	30 (73.2%)	27 (81.2%)	5 (71.4%)	
**Current status**					**p = 0.60**
In remission	21 (30.1%)	14 (34.1%)	11 (33.3%)	3 (42.9%)	
Not in remission	39 (57.4%)	19 (46.3%)	20 (60.6%)	3 (42.9%)	
Deceased	8 (11.8%)	7 (17.1%)	1 (3.03%)	1 (14.3%)	

^a^ZIP codes were used to group patients into quartiles based on median annual household income, with quartile 1 earnings $0 - $18,900, quartile 2 earnings $19,000 – 32,800, quartile 3 earnings 32,900 – 56,000, quartile 4 earnings $57,000 – 130,300

^b^Other cancer types included lymphoma (Hodgkin’s, T-cell, large cell), multiple myeloma, esophageal cancer, gastric cancer, abdominal desmoid cancer, carcinoid tumor, bladder cancer, ovarian cancer, endometrial cancer, leiomyosarcoma, metastatic cancer with an unknown primary

^c^Decrease in EF defined by symptomatic reduction in EF by at least 5% to less than 55% or an asymptomatic reduction in EF by at least 10% to less than 55% according to Cardiac Review and Evaluation Committee of Trastuzumab-associated Cardiotoxicity

### Clinical characteristics

**Fig. 2:**
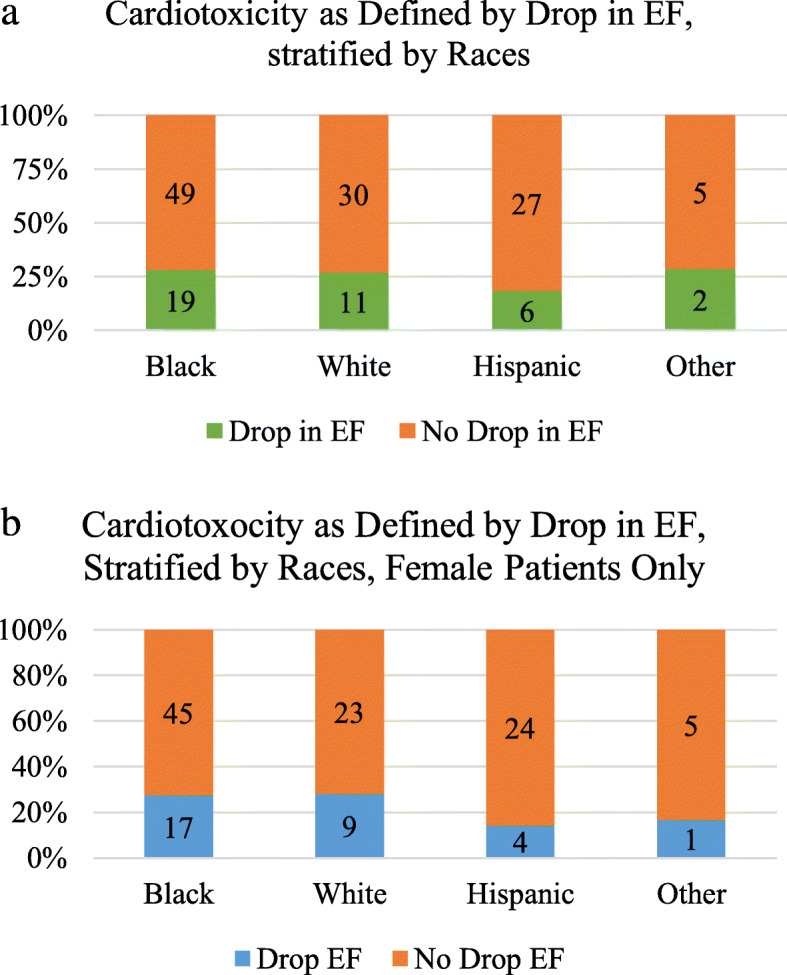
Cardiotoxicity as Defined by Drop in EF. **a** Comparing between the racial groups, there is no statistical difference in the number of patients who decrease in EF: 19 [27.9%] blacks, 11 [26.8%] whites, 6 [18.2%] ispanics, 2 [28.6%] other races, *p* = 0.75. **b** When only looking at female patients, there remains to be no statistical difference in the number of patients who had a decrease in EF: 17 [25.0%] blacks, 9 [28.1%] whites, 4 [14.3%]. Hispanics, 1 [16.7%] other races, p = 0.51

There was no statistical difference between racial groups in comorbidities, procedures, medication used prior to chemotherapy initiation, cancer stage at diagnosis, and use of trastuzumab versus doxorubicin (Table 1). Breast cancer was the most common cancer type, with “other types” being very broad, including lymphoma (Hodgkin’s, T-cell, large cell), multiple myeloma, esophageal cancer, gastric cancer, abdominal desmoid cancer, carcinoid tumor, bladder cancer, ovarian cancer, endometrial cancer, leiomyosarcoma, and metastatic cancer with unknown primary. White patients were less likely to have breast cancer in this cohort (24 [58.5%], compared to 57 [83.8%] black patients, 24 [72.7%] Hispanic patients, and 5 [71.4%] other race patients, *p* = 0.036). There was no statistical difference when comparing racial groups in the number of patients who had drops in EF (19 [27.9%] blacks, 11 [26.8%] whites, 6 [18.2%] Hispanics, 2 [28.6%] other races, *p* = 0.75) (Fig. 2a). There was no difference in the number of patients whose EF dropped below 40% (*p* = 0.36). When looking only at female patients, there was still no difference in drop in EF (*p* = 0.51) (Fig. 2b). The number of patients in remission was also similar between racial groups (*p* = 0.60).

### Referral pattern

A total of 70 (47.0%) patients were referred by oncology clinics to cardio-oncology clinic or general cardiology clinic (50 [71.4%] to cardio-oncology and 20 [28.6%] to general cardiology clinic). When stratified by race, no group was less likely to be referred (33 [48.5%] blacks, 21 [51.2%] whites, 14 [42.4%] Hispanics, 2 [28.6%] others, *p* = 0.66) (Table 2). The number of oncology appointments attended was similar between groups (*p* = 0.29). The number of echocardiograms received since starting cardiotoxic chemotherapy was similar between groups (4 for blacks, 4 for whites, 5 for Hispanics, 4 for others, *p* = 0.62). Among those who were referred to cardio-oncology or cardiology clinic, the median number of appointments attended was 4 (IQR 2–6) and median number of appointments missed or cancelled was 2 (IQR 0–4), with no difference between racial groups (*p* = 0.88, *p* = 0.20, respectively). More patients with EF were referred to cardiology clinic (32 referred vs 6 non-referred low EF patients, *p* < 0.0001). These 6 non-referred patients all had either metastatic cancer or were determined to be too frail to tolerate a different chemotherapy regimen and were referred for hospice. When stratified by race, similar number of patients received additional cardiac imaging tests (Table 3). Black patients were more likely to receive angiotensin converting enzyme inhibitor (ACEI) / angiotensin II receptor blocker (ARB) post chemotherapy initiation (*p* = 0.047). When stratified by referral, again similar number of patients received additional cardiac imaging tests. Referred patients were more likely to receive beta blocker, ACEI/ARB, hydralazine, mineralocorticoid antagonist (MRA), and statin (Table 4).

**Table 2:** Clinical characteristics of patients regarding access to care by race

All Patients	Black (N = 68)	White (N = 41)	Hispanic (N = 33)	Other (N = 7)	*P*-value
**Referred to cardiology clinic**	33 (48.5%)	21 (51.2%)	14 (42.4%)	2 (28.6%)	***p = 0.66***
**Number of oncology appointments attended (median [IQR])**	24 (12, 32)	17 (13, 24)	21 (15, 28)	16 (13, 17)	***p = 0.29***
**Number of echocardiograms (median [IQR])**	4 (3, 7)	4 (2, 6)	5 (3, 7)	4 (3, 5)	***p = 0.62***
**Referred Patients**	Black (N = 33)	White (*N* = 21)	Hispanic (*N* = 14)	Other (N = 2)	*P*-value
**Number of cardio-oncology or cardiology appointments attended (median [IQR])**	4 (2, 6)	5 (2, 9)	4 (4, 5)	4 (3, 5)	***p = 0.88***
**Number of cardio-oncology or cardiology appointments missed or cancelled (median [IQR])**	1 (0, 3)	3 (1, 5)	2 (1, 4)	1 (0, 2)	***p = 0.20***
**Number of echocardiograms in referred patients (median [IQR])**	6 (4, 8)	5 (2, 7)	6 (5, 7)	5 (4, 6)	***p = 0.76***

*IQR* interquartile range

**Table 3:** Imaging and medications of patients regarding access to care, by race

	Black (N = 68)	White (N = 41)	Hispanic (N = 33)	Other (N = 7)	***P***-value
**Number of patients with additional cardiac imaging**					
MUGA scan	8	3	5	0	***p*** **= 0.55**
Cardiac CT/MRI	2	3	1	0	***p*** **= 0.63**
Stress test	13	9	8	1	***p*** **= 0.89**
RHC	3	3	0	0	***p*** **= 0.42**
LHC	4	3	1	0	***p*** **= 0.78**
**Medication post-chemo**					
Beta blocker	38	20	14	7	***p*** **= 0.12**
ACEI/ARB	43	18	12	3	**p = 0.047**
Diuretic	20	13	6	1	**p = 0.54**
CCB	20	6	4	2	**p = 0.13**
Hydralazine	5	2	0	0	**p = 0.38**
Nitrate	2	3	0	0	***p*** **= 0.33**
MRA	2	4	1	1	***p*** **= 0.28**
Antiarrhythmic	2	2	0	0	**p = 0.60**
Aspirin	19	15	7	3	***p*** **= 0.43**
Statin	24	16	11	3	***p*** **= 0.94**

**Table 4:** Imaging and medications os patients regarding access to care, by referral status

	Referred (***N*** = 70)	Not Referred (***N*** = 79)	***P***-value
**Number of patients with additional cardiac imaging**			
MUGA scan	7	16	***p*** **= 0.11**
Cardiac CT/MRI	4	2	**p = 0.42**
Stress test	11	7	***p*** **= 0.22**
RHC	2	4	***p*** **= 0.68**
LHC	1	7	***p*** **= 0.07**
**Medication post-chemo**			
Beta blocker	54	24	**p < 0.0001**
ACEI/ARB	50	26	**p < 0.0001**
Diuretic	22	18	***p*** **= 0.23**
CCB	14	18	**p = 0.68**
Hydralazine	6	1	***p*** **= 0.035**
Nitrate	4	1	**p = 0.13**
MRA	7	1	***p*** **= 0.018**
Antiarrhythmic	2	0	**p = 0.49**
Aspirin	24	20	**p = 0.23**
Statin	32	22	***p*** **= 0.024**

**Fig. 3:**
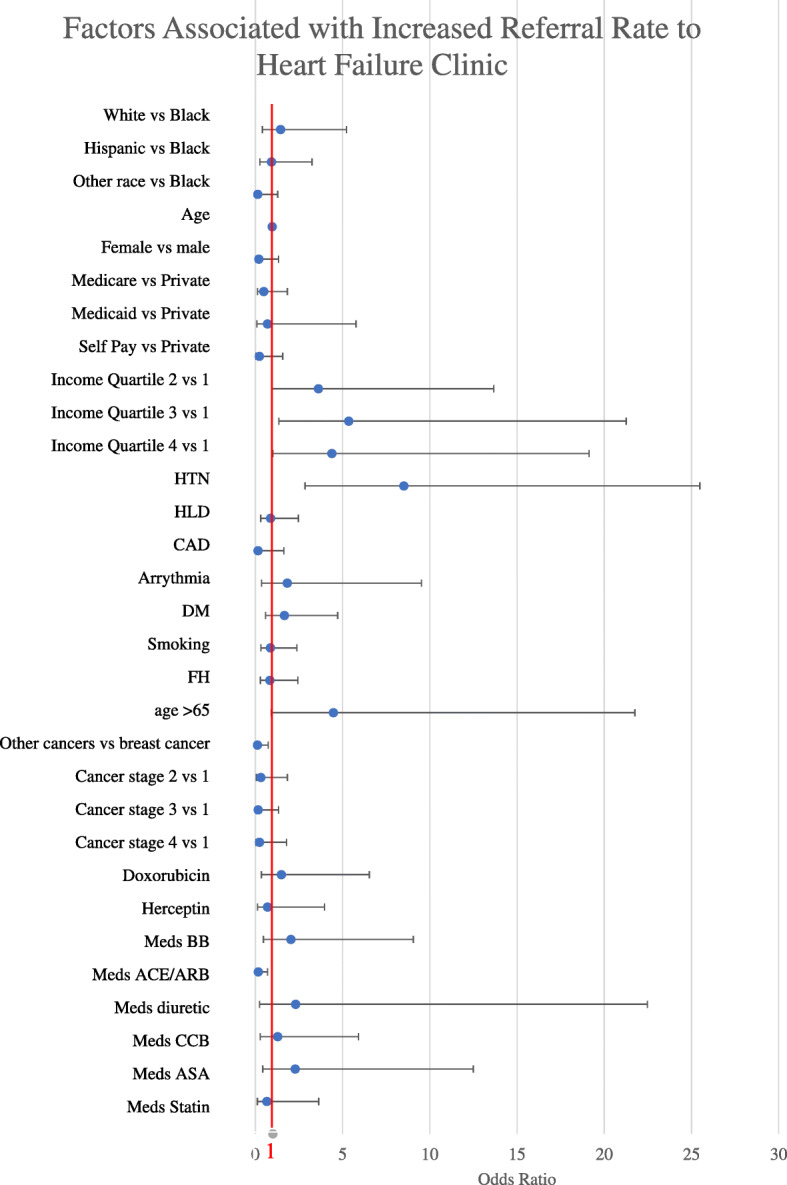
Factors Associated with Increased Referral Rate to Heart Failure Clinic

In unadjusted univariate analysis, patients were more likely to be referred if they lived in ZIP codes with median household income quartiles 2–3, were hypertensive, had coronary artery disease, had arrythmia, had breast cancer, or received trastuzumab for chemotherapy (Table 5). A logistic regression model used race, age, gender, insurance, income quartile by home ZIP code, comorbidities (HTN, HLD, CAD, arrythmia, DM, smoking, FH, age > 65), medications pre-chemo (beta blocker, ACEI/ARB, diuretic, CCB, ASA, statin), cancer type, cancer stage, and chemotherapy to look at association to referral rate (Table 6). Hydralazine, nitrate, MRA, and antiarrhythmic were not included in the model due to extremely low number of patients taking these medications prior to chemotherapy intiaiton. This model found that increased referral rate was best explained by income quartile by income quartile 3 (*p* = 0.017), income quartile 4 (*p* = 0.049), hypertension (*p* = 0.0001), and cancer type (*p* = 0.022). Compared to patients in income quartile 1 as reference, quartile 3 patients were 5.35 times more likely to be referred (CI 1.25–21.26, p = 0.017), quartile 4 patients were 4.38 times more likely to be referred (CI 1.00–19.13, p = 0.049). Quartile 2 patients were not statistically more likely to be referred, however the confidence interval pattern does trend towards increased referral as well (OR 3.61, CI 0.95–13.67, *p* = 0.06) (Fig. 3). Patients who were hypertensive were more likely to be referred (OR 8.51, CI 2.84–25.38, *p* < 0.0001). Patients with non-breast cancers were less likely to be referred (OR 0.12, CI 0.02–0.74, *p* = 0.02). Other comorbidities such as HLD, CAD, arrythmia, DM, smoking, and family history were not associated with increased referral rate. Race, age, gender, insurance, cancer stage, and type of chemotherapy were not associated with increased referral rate. We point out that we do see the phenomenon of multicollinearity here: ACEI/ARB is shown to be associated with less referral despite an insignificant association on the binary model in Table 5. This association disappears when “hypertension” is removed from the multiple regression model. The opposite is not true: when “hypertension” is included and “ACEI/ARB” is not, “hypertension” remains positively associated with referral. Multicollinearity in this case does not reduce the predictive power of the model as a whole, however does affect calculations regarding the individual predictor “hypertension”.

**Table 5:** Referral patterns of patients receiving cardiotoxic chemotherapy agents from oncology clinic to cardio-oncology or cardiology clinic

	Referred (N = 70)	Not Referred (N = 79)	*P*-value
**Race**			**p = 0.66**
Black	33 (47.1%)	35 (44.3%)	
White	21 (30.0%)	20 (25.3%)	
Hispanic	14 (20.0%)	19 (27.9%)	
Others	2 (2.9%)	5 (6.3%)	
**Age at diagnosis (median, [IQR])**	61 (51, 68)	56 (50, 64)	**p = 0.55**
**Gender**			**p = 0.68**
Male	9 (12.9%)	12 (15.2%)	
Female	61 (87.1%)	67 (84.8%)	
**Insurance**			**p = 0.18**
Private insurance	39 (55.7%)	39 (49.4%)	
Medicare	26 (37.1%)	25 (31.6%)	
Medicaid	2 (2.9%)	3 (3.8%)	
No insurance	3 (4.3%)	12 (15.2%)	
**Income by ZIP code**			***p*** **= 0.039**
Quartile 1	11 (15.7%)	26 (32.9%)	
Quartile 2	17 (24.3%)	18 (22.8%)	
Quartile 3	23 (32.9%)	15 (19.0%)	
Quartile 4	19 (27.1%)	20 (25.3%)	
**Risk factors**			
HTN	52 (74.3%)	32 (40.5%)	**p < 0.0001**
HLD	25 (35.7%)	23 (29.1%)	***p*** **= 0.39**
CAD	12	0	***p*** **= 0.0006**
Arrhythmia	11	4	**p = 0.035**
DM	22 (31.4%)	17 (21.5%)	***p*** **= 0.17**
Smoking	27 (38.6%)	34 (42.0%)	***p*** **= 0.67**
FH	20 (28.6%)	20 (24.7%)	***p*** **= 0.59**
Age > 65	20 (28.6%)	14 (17.7%)	**p = 0.12**
**Procedures**			
Coronary stents	4	0	**p = 0.13**
Cardiac surgery	1	0	***p*** **= 1.00**
**Medication pre initiation of chemotherapy**			
Beta blocker	13	12	**p = 0.63**
ACEI/ARB	26	19	***p*** **= 0.08**
Diuretic	5	5	***p*** **= 0.84**
Hydralazine	1	1	***p*** **= 0.93**
Nitrate	1	1	**p = 0.93**
MRA	0	0	**p = 1**
CCB	8	9	***p*** **= 0.99**
Antiarrhythmic	0	0	**p = 1**
Aspirin	14	10	**p = 0.22**
Statin	17	11	**p = 0.11**
**Cancer type**			***p*** **= 0.005**
Breast cancer	59 (84.3%)	52 (64.3%)	
Other cancer	11 (15.7%)	29 (35.8%)	
**Cancer stage**			**p = 0.07**
Stage 1	8 (11.4%)	4 (4.9%)	
Stage 2	38 (54.3%)	33 (40.7%)	
Stage 3	11 (15.7%)	18 (22.2%)	
Stage 4	13 (18.6%)	26 (32.1%)	
**Chemotherapy**			**p = 0.036**
Doxorubicin	22 (31.4%)	42 (51.9%)	
Herceptin	40 (57.1%)	34 (42.0%)	
Doxorubicin and herceptin	8 (11.4%)	5 (6.2%)

**Table 6:** Multivariate analysis of variables associated with increased referral rate of patients receiving cardiotoxic chemotherapy from oncology clinic to cardio-oncology or cardiology clinic

	Odds Ratio	Confidence Interval	***P***-value
**Race**			
Black	Reference		
White	1.47	0.50–4.32	***p*** **= 0.48**
Hispanic	1.34	0.43–4.19	**p = 0.62**
Other	0.42	0.05–3.74	**p = 0.43**
**Age**	0.96	0.93–1.00	**p = 0.08**
**Gender**			
Male	Reference		
Female	0.20	0.03–1.33	***p*** **= 0.10**
**Insurance**			
Private	Reference		
Medicare	0.48	0.13–1.84	**p = 0.29**
Medicaid	0.69	0.08–5.77	***p*** **= 0.74**
Self	0.23	0.03–1.57	**p = 0.13**
Income by ZIP code*			
Quartile 1	Reference		
Quartile 2	3.61	0.95–13.67	**p = 0.06**
Quartile 3	5.35	1.35–21.26	**p = 0.017**
Quartile 4	4.38	1.00–19.13	**p = 0.049**
**Risk factors**			
HTN	6.85	2.68–17.52	**p = 0.0001**
HLD	1.07	0.41–2.81	***p*** **= 0.90**
CAD	0.15	0.01–1.63	**p = 0.12**
Arrhythmia	1.82	0.35–9.52	**p = 0.48**
DM	1.78	0.67–4.75	***p*** **= 0.25**
Smoking	1.18	0.47–2.99	***p*** **= 0.73**
FH	0.79	0.29–2.13	***p*** **= 0.64**
Age > 65	4.01	0.10–16.17	***p*** **= 0.051**
**Cancer type**			
Breast cancer	Reference		
Other cancer^	0.12	0.02–0.74	**p = 0.02**
**Cancer stage**			
Stage 1	Reference		
Stage 2	0.31	0.05–1.83	**p = 0.20**
Stage 3	0.16	0.02–1.33	***p*** **= 0.09**
Stage 4	0.23	0.03–1.78	***p*** **= 0.16**
**Chemotherapy**			
Doxorubicin	1.49	0.34–6.53	**p = 0.60**
Herceptin	0.70	0.12–3.97	**p = 0.68**
**Medications pre-chemotherapy**			
BB	2.04	0.46–9.06	**p = 0.35**
ACEI/ARB	0.16	0.04–0.70	**p = 0.01**
Diuretic	2.31	0.24–22.47	***p*** **= 0.47**
CCB	1.28	0.28–5.91	**p = 0.75**
ASA	2.28	0.42–12.49	***p*** **= 0.34**
Statin	0.66	0.12–3.63	**p = 0.63**

## Discussion

Health care disparities in medicine have become an increasingly important topic in today’s field of practice as we learn each day about its impact on the way we practice medicine and alter patient outcomes. Over the past decade, we have made many strides in providing patients with optimal cardiovascular care. However, despite our best efforts, health care disparities remain far from being ameliorated. Black patients continue to have a shorter life expectancy with 32–43% of the mortality difference being due to cardiovascular disease [18]. Studies looking at health care disparities in the cardio-oncology population are lacking. Despite our population of black patients living predominantly in ZIP codes associated with lower income, we found no statistical significant difference in access to care or clinical outcomes. This is highlighted by the similar number of attended appointments, cancelled appointments, echocardiograms received, proportion of patients who had a decrease in EF, current remission status, number of patients receiving additional cardiac imaging, and medications post chemotherapy. One prior study showed that black female patients had higher rates of cardiotoxicity from trastuzumab compared to white female patients [19, 20]. Our study looked at both sexes and included both trastuzumab and anthracyclines, and found no correlation between race and rates of cardiotoxicity.

Due to lack of established referral guidelines, the decision for oncologists to refer patients to cardiologists can be varied. For example, while some suggest referral only if there are signs and symptoms of cardiac dysfunction, others suggest referral anytime a patient has EF < 40% or EF 40–55% if EF remains depressed after withholding therapy for 6–8 weeks [19, 20]. These variations in the referral process are unfortunately common scenarios and therefore a potential source of health care disparities. Our study was the first study to compare referral patterns among racial groups in the cardio-oncology population, and we found no difference among racial groups. As our hospital is a safety net hospital with a population vulnerable to health care disparities, these findings were initially reassuring. The logistic regression model then revealed that patients from higher median income by ZIP codes were associated with higher referral rates despite these patients having similar cardiovascular risk factors and similar changes in EF compared to the rest of the population from our study. Other studies have shown similar results in the heart failure population: heart failure patients of higher socio-economic score (SES) were more likely to be referred to palliative care, while new heart failure patients with lower SES had significantly longer times to cardiology consultation [21, 22]. The identification of such referral biases leaves room for much improvement. Proposed methods can include standard referral guidelines and implementation of multidisciplinary clinics.

The nuances of chemotherapy and cardiotoxicity can be a complex process; while oncologists are not well versed in cardiovascular complications, general cardiologists may not be familiar with the specific nuances of each chemotherapy agent. There are currently no standardized national or international guidelines for oncologists and cardiologists to follow for how to go about managing cardiotoxicity and when to refer patients to cardiology, as evidenced by the differing TTE monitoring guidelines between different societies [23]. For example, the American Society of Echocardiography recommends TTE monitoring every 3 months during trastuzumab therapy and evaluation before each cycle for anthracycline doses > 240 mg/m^2^, whereas the American Society of Clinical Oncology recommends 6–12 month post-treatment echocardiogram monitoring, and any additional frequency of cardiac imaging as determined by clinical judgment and patient circumstances.

Additionally, the definition of cardiotoxicity in the majority of literature has been regarding chronic cardiotoxicity, manifested by symptoms of heart failure or decrease in EF on echocardiogram. Acute and subacute cardiotoxicities are much more difficult to be monitored and caught by oncologists and oftentimes are more ideally managed by cardiologists. These are typically electrical abnormalities that are manifested by palpitations or subtle changes on electrocardiogram. Although not as significant a cause of morbidity and mortality as the typical cardiomyopathy we see from cardiotoxicity, these mechanisms can oftentimes be predisposing factors and early manifestations of impending cardiac disease.

In light of these challenges, cardio-oncology programs involving multidisciplinary approaches have rapidly emerged with the common goal of preserving cardiac function and helping patients safely finish chemotherapy treatments. A survey conducted by the American College of Cardiology (ACC) distributed to centers with cardiology programs found that only 27% of centers had dedicated cardio-oncology programs [24]. An observational study of a multidisciplinary clinic demonstrated that despite 55% of patients experiencing a decrease of EF by at least 10, 81% of the patients were able to complete greater than 90% of trastuzumab therapy [25]. A similar study of a larger patient population found that 191 of the 225 patients (85.3%) were able to complete their prescribed cancer treatments [26].

It is uncertain whether such trends will apply to other states in the United States. In previous papers specifically looking at racial disparity among breast cancer patients or heart failure patients in different geographical regions, the studied outcomes were similar across different cities and states [27–29]. This study does provide some reassurance that racial differences do not affect patient access or patient care in the urban areas of Philadelphia.

Several limitations exist in this study. First, data was combined for patients receiving trastuzumab and anthracyclines. However, each drug has a different mechanism of toxicity and can vary in patient profile and risk factors. The definition of cardiotoxicity also varies greatly across different societies. Selection bias does exist in our population as those who seek out care for chemotherapy are presumed to have better medical literacy at baseline. The presence of a cardio-oncology program in our hospital may also play a role in the outcomes seen. We used ZIP codes as a surrogate to create income quartiles, a common practice in many SES analyses. In fact, home ZIP codes can be associated with other factors such as education, medical literacy, family support, etc. that can account for the trends seen here. Lastly, the retrospective nature of this manuscript is a limitation. The findings in this manuscript should be further explored with prospective studies to test validity of the results and applicability outside of the institution.

## Conclusion

The results of this study suggest that patients of our cardio-oncology population at a safety net hospital receive the same level of surveillance and treatment, and achieve similar clinical outcomes in cardiotoxicity regardless of race. However, patients with home ZIP codes with higher income quartiles, hypertension, and breast cancer do trend towards increased likelihood of referrals to cardio-oncology clinics. We also emphasize the importance of establishing and adhering to standardized protocols in cardiotoxocity monitoring.

## Data Availability

The dataset analyzed during the current study is available from the corresponding author upon reasonable request.

## Abbreviations

ACEI/ARB: Angiotensin-converting enzyme inhibitor / angiotensin receptor blocker
CAD: Coronary artery disease
CCB: Calcium channel blocker
DM: Diabetes mellitus
EF: Ejection fraction
FH: Family history (of heart failure)
HTN: Hypertension
HLD: Hyperlipidemia
HER: Human epidermal growth factor receptor
IQR: Interquartile range
MRA: Mineralocorticoid receptor antagonist
SES: Socioeconomic status
TTE: Transthoracic echo
ZIP: Zone improvement plan
